# Knowledge, Attitude, and Psychological Impacts of COVID-19 in Saudi Arabia

**DOI:** 10.3389/fpubh.2022.801777

**Published:** 2022-02-23

**Authors:** Naif Alanazi, Khaled Bahjri

**Affiliations:** ^1^Department of Public Health, College of Health Science, Saudi Electronic University, Riyadh, Saudi Arabia; ^2^Department of Pharmaceutical and Administrative Sciences, Loma Linda, CA, United States

**Keywords:** COVID-19 knowledge, attitude, mental health perceptions, coronvirus-2019 stress and anxiety, knowledge and attitude

## Abstract

**Background:**

The world has been facing an unprecedented pandemic of COVID-19 with over 336 million people infected and millions of deaths. This required an enormous communication effort response from governments, international, and individuals to keep the public informed about the outbreak. When a pandemic affects communities, individuals' knowledge and attitude are important factors to contain the outbreak. Thus, the purpose of this study is to measure individuals' knowledge and attitude toward COVID-19 and ascertain whether a need exists for mental health services for those who were affected by the pandemic.

**Methods:**

A cross-sectional design was used to measure the knowledge, attitude, and psychological impacts about the COVID-19 pandemic among the Saudi population. Research participants were recruited using a snowball sampling technique through the social media platform WhatsApp. A total of 482 eligible individuals participated from various locations in Saudi Arabia represented almost all Saudi regions. The questionnaire consisted of seven questions evaluating knowledge, seven gauging attitudes, and 16 questions assessing anxiety and perceived need for mental healthcare services.

**Results:**

A modest level of knowledge (59%) was found among the Saudi population sampled about the COVID-19 pandemic, and satisfactory knowledge (>80%) about its preventive measures. Anxiety and stress existed among the participants (79% obsessed with COVID-19), with an 88% approval rate for obtaining mental health services for individuals highly affected by the pandemic.

**Conclusion:**

There is a need to increase awareness and provide continuous updates regarding the pandemic situation. Promoting access to mental health services is critical, as well as finding creative and suitable strategies to deliver mental health services to those who need them.

## Introduction

The family of coronaviruses has been responsible for three outbreaks in the last 20 years. The earliest pandemic, severe acute respiratory syndrome coronavirus (SARS-CoV) occurred in China 2002–2003 (1), followed by Middle East respiratory syndrome coronavirus (MERS-CoV), which surged in Saudi Arabia in 2012 (2). The worst pandemic in our lifetime emerged in 2019 in Wuhan, China, widely known as COVID-19. This novel coronavirus was named by the International Committee on Taxonomy Virology (ICTV) as severe acute respiratory syndrome coronavirus-2 (SARS-CoV-2) (3). COVID-19 differs from other coronaviruses in terms of its high transmissibility from person to person, and pathological shedding, which may persist longer than SARS-CoV and MERS-CoV (4). As a result, on March 11, 2020, the World Health Organization (WHO) identified COVID-19 as a global pandemic.

As of January 2022, there were ~336 million confirmed cases of COVID-19, with over 5.5 million confirmed deaths worldwide (5). These numbers are expected to increase as new variants of the virus surge around the world. In fact, COVID-19 has had a wider effect than any virus in its genus. In addition, COVID-19 has impacted all aspects of life worldwide. Its surge and rapid spread have caused chaos, anxiety, and a multitude of concerns at the local, national, and international levels. Social and economic activities have been curtailed by lockdowns and quarantine measures, initiating a series of adverse economic, public health, and environmental consequences (6). The virus has changed the world significantly, taking a significant toll on individuals, families, and communities (7). Fundamental changes have been seen in our social, communication, thoughts, working, and even physical appearance.

The world continues to deal with the effects of the pandemic as new variants emerge and spread. High rates of infection and a rising death toll creates pressure on global governments to collaborate on ways to contain the outbreak. This in turn places a huge responsibility on individuals, communities, and governments to limit further health, social, and economic devastation caused by the pandemic. While governments are leading large-scale efforts toward virus containment, in fact, it is individuals who are the cornerstone of the containment efforts of this pandemic (8). It is likely that individuals' knowledge and attitudes toward COVID-19 explains the speed of transmission in different societies. Individuals' health literacy and attitude toward the virus symptoms and modes of transmission and, use of personal protection equipment can play a role in the rate of infection. Individuals can protect themselves, their families, and communities by social distancing, wearing face masks, washing hands frequently, and most importantly getting vaccinated. Mathematical models conducted exclusively in the Saudi population to predict biological and epidemiological predispositions toward COVID-19 found that social distancing, personal hygiene, and travel restrictions were vital measures to prevent the outbreak's spread (9). Evaluating whether individuals aware, embrace, or ignore these measures can provide authorities with insight into how open a given community or region is to adopting measures to deal with the pandemic. Evidence-based data about knowledge and attitudes of COVID-19 outbreak can help health officials to develop effective interventions and policies that are appropriate to a particular population (10). Several studies have shown that individuals lack knowledge about microbial infections in the general population. A lack of community knowledge about disease transmission can impede efforts to control the pandemic (11, 12).

Knowledge and attitudes toward COVID-19 prevention behaviors can be explained by many factors, including gender, educational level, geographical area, socioeconomic status, access to healthcare services, and level of pandemic burden. Generally, when a pandemic hits, knowledge and attitude levels are usually not aligned with the protective actions needed to slow or stop transmission. In order to understand human behaviors, it is crucial to know the knowledge and attitude when people take actions for themselves (13). Therefore, the purpose of this study is to measure individuals' knowledge and attitude toward COVID-19 to know the effectiveness of the protective actions taken by health officials in Saudi Arabia. Furthermore, it is also likely that the pandemic will cause adverse psychological impacts across society in general. The study of individuals' anxiety and stress may help health officials effectively manage the pandemic and understand the mental health status of the population. This may turn health authorities' attention to providing mental health services for those adversely affected by the pandemic (13). As a result, this study sought to measure the level of anxiety associated with the COVID-19 outbreak and provide insight into the perceived mental health needs among the target population of Saudi citizens.

## Methods

A cross-sectional design study was used to measure the knowledge and attitudes that exist in Saudi population regarding COVID-19. A total of 482 individuals responded to the online survey. Research participants were recruited using the snowball sampling technique through WhatsApp, one of the most popular social media platforms in Saudi Arabia. The self-administered, online survey was developed using Qualtrics XM software, Version 2020. A consent form was attached, which participants must complete before beginning the survey. A hyperlink to the survey was distributed through WhatsApp to all groups and contacts of the principal investigator, who were encouraged to share it with their own contacts. By clicking on the link, participants would be directed first to the consent form, which they must complete in order to access the research survey.

The survey was provided in both English and Arabic, translated from English to Arabic using Brislin's model (14, 15). Eligible participants were 16 years old or older, and able to read either Arabic or English. The data collection process was initiated on March 2021 until the end of May of 2021; data were gathered from various provinces in Saudi Arabia. After starting the survey, participants could stop at any time and complete it at their convenience.

The instrument used in this study was a modified version of one used in a similar study conducted in India (16). The survey contained five sections. The first section focused on socio-demographic variables including age, gender, marital status, occupation, education level, and area of residence. The second section dealt with COVID-19 awareness (knowledge) with seven multiple choice questions. The third part contained seven 5-point Likert style questions (ranging from strongly agree, agree, do not know, disagree, and strongly disagree) about participants' attitudes about COVID-19. Anxiety regarding COVID-19 was assessed by 13 questions using a 5-point Likert scale (ranging from never, occasionally, sometimes, often, and always). The perception of healthcare need was measured by four 3-point Likert scale questions (yes, maybe, and no).

## Results

### Participants' Characteristics

**Figure 1:**
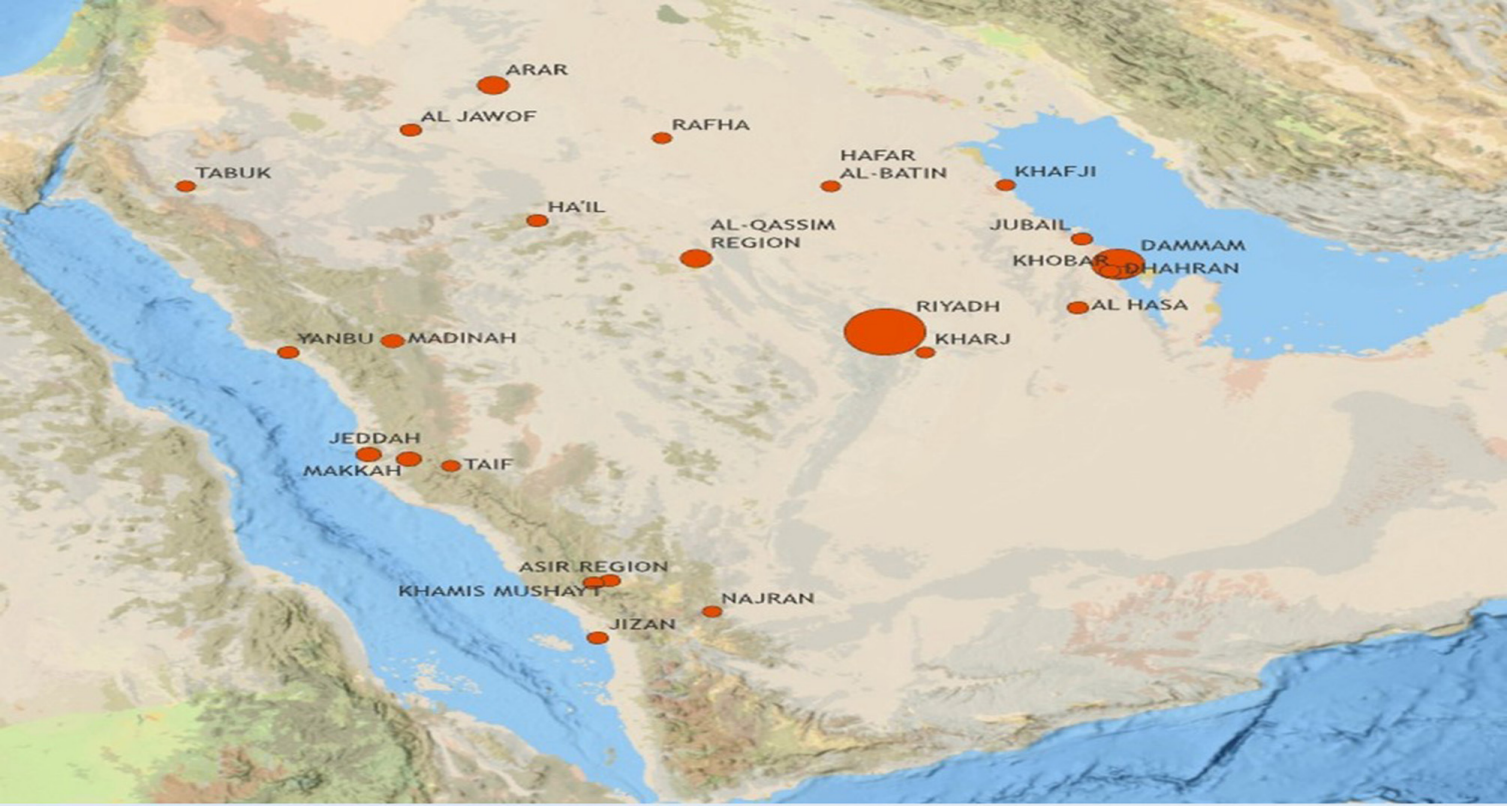
Distribution of study sample across Saudi Arabia.

A total of 482 eligible individuals participated from various locations in Saudi Arabia represented almost all Saudi regions. The high participation rate came from populous cities such as Riyadh, Dammam, Al-Qassim, Jeddah, and Makkah as it is shown in Figure 1.

From the sample collected, 62 participants were found to be under age 16 and therefore excluded from the study. Most of the study participants were young, with the median age ~23 years old. The number of male participants (53%) were slightly higher than female participants (47%). A majority of participants (66%) were married, and more than 60% held a bachelor's degree or higher. Only 12% of participants were in the healthcare field. More demographic details can be found in Table 1.

**Table 1:** Subject characteristics (*N* = 482).

**Age; median (min. to max.)**		**23 (16–58)**	
Gender	Male	252	53%
	Female	227	47%
Marital status	Single	140	30%
	Married	314	66%
	Divorced	16	3%
	Widowed	3	1%
Occupation	Professional	129	27%
	Business	13	3%
	Student	74	15%
	Healthcare	59	12%
	Unemployed	78	16%
	Others	127	26%
Education	^*^HS or less	99	21%
	Diploma	81	17%
	Bachelors	224	47%
	Masters	47	10%
	Doctorate	28	6%

*All values are count and percentage except otherwise indicated*.

* *HS means high school*.

### Awareness of COVID-19 Outbreak

Awareness of COVID-19 was found to be moderately high among the study population, as shown in Tables 2–4. Respondents reported essential knowledge of the virus, with most (59%) believing that COVID-19 was spread through multiple modes such as sneezing, touching, kissing, and ingesting food (see Table 2).

**Table 2:** How does COVID-19 (Coronavirus Disease-2019) spread?

	***N***	**%**
Touching	181	38%
Sneezing	191	40%
Kissing	169	35%
Food	31	6%
All the above	285	59%

However, a small margin of the participants (6%) thought the virus was spread solely by food. A significant number of participants recognized that handwashing, avoiding indoor crowds, using face coverings, and social distancing could stop dissemination of the virus. The vast majority of the study participants (90%) believed that isolating a person with symptoms could stop the spread of the infection. In addition, most of the participants (>70%) considered the virus highly contagious and untreatable (see Table 3).

**Table 3:** COVID-19 infection knowledge.

	***N***	**%**
**How does COVID-19 spread?**		
Highly contagious	345	72%
Highly fatal	72	15%
No treatment	350	73%
Transmission cannot be prevented	75	16%
Self-quarantine is ineffective	48	10%
COVID-19 spread score (mean ± standard deviation)	1.8 ± 1.1	
**Which of the following can protect you from COVID-19?**		
Handwashing	404	84%
Avoiding indoor crowds	399	83%
Face covering and social distancing	404	84%
Touching nose and eyes	74	15%
Reusing masks and gloves	64	13%
Shaking hands	62	13%
COVID-19 protection score (mean ± standard deviation)	2.9 ± 1.2	

**Table 4:** What are the symptoms of COVID-19?

	***N***	**%**
Fever/chills	381	79%
Cough	291	60%
Shortness of breath	421	87%
Fatigue	189	39%
Muscle ache	294	61%
Headache	336	70%
Loss of taste or smell	373	77%
Sore throat	252	52%
Congestions	153	32%
Nausea/vomiting	143	30%
Diarrhea	231	48%

**Figure 2:**
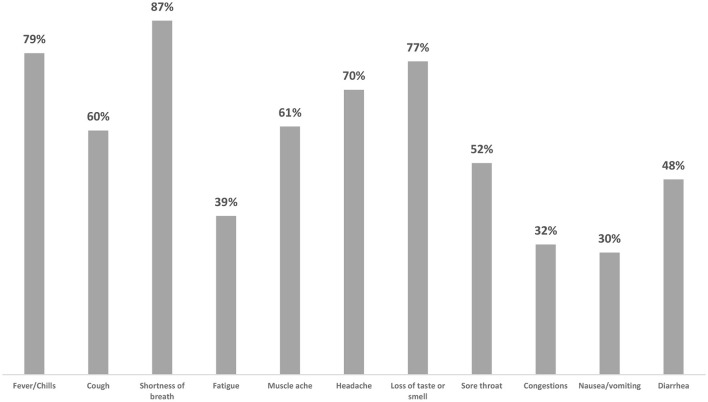
What are the symptoms of COVID-19?

Furthermore, participants were able to identify major COVID-19 symptoms such as fever/chill, shortness of breath, cough, loss of taste/smell, and headache. Nevertheless, fewer than half of participants were less aware of symptoms such as fatigue, congestions, nausea/vomiting, and diarrhea as shown in Table 4 and Figure 2.

### Attitude Toward COVID-19

A significant number of participants (89%) felt that they could be infected by COVID-19; in addition, a super-majority (95%) agreed with government guidelines and regulations regarding the pandemic. Respondents thought frequent hand-washing and social distancing could slow the spread of the virus, and more than 96% said they were likely to quarantine/isolate themselves if they had fever and cough. Nevertheless, more than half of participants (53%) thought traveling within the country was safe during the pandemic. In addition, 51% believed that patients who recovered after being infected with the virus should not attend indoor activities or social gatherings. More details of participant attitudes toward COVID-19 can be found in Table 5.

**Table 5:** Attitude toward COVID-19 infection.

	**Never**	**Unlikely**	**Maybe**	**Most likely**	**Definitely**
Do you feel you can be affected by the coronavirus?	15 (3%)	37 (8%)	197 (41%)	186 (39%)	42 (9%)
Would you agree to the government guidelines regarding the coronavirus pandemic?	17 (4%)	7 (1%)	22 (5%)	227 (47%)	206 (43%)
Do you think washing hands frequently can lower the risk of coronavirus infection?	13 (3%)	10 (2%)	42 (9%)	219 (46%)	196 (41%)
How likely are you to quarantine/ isolate yourself if you have fever and cough?	10 (2%)	4 (1%)	48 (10%)	224 (47%)	193 (40%)
Do you think social distancing is essential to stop spread of the coronavirus?	11 (2%)	10 (2%)	17 (4%)	188 (39%)	254 (53%)
Do you think traveling across/within the country is safe during these times?	81 (17%)	147 (31%)	86 (18%)	123 (26%)	43 (9%)
Patients who are cured from corona virus should not attend indoor activity or social gathering?	85 (18%)	145 (30%)	111 (23%)	97 (20%)	40 (8%)

### Anxiety of COVID-19 Pandemic

As the data of the Table 6 showed, ~79% of respondents (those who answered *maybe, most likely*, or *definitely*) were obsessed with COVID-19 over the previous week. Nearly 57% of respondents felt paranoid about contracting the coronavirus. About 82% of participants said they were likely to avoid partying, meeting, and gathering, and 77% reported they were likely to avoid social contact. COVID-19 was the topic that most participants (76%) talked about with their friends over the last week. Furthermore, about 28% of the participants reported having difficulty sleeping due to worries about the pandemic. However, most participants (68%) did not often feel the need to buy and stock essentials at home. More than half of participants (54%) felt scared if anyone in their social circle was sick with something that might be COVID-19.

**Table 6:** Anxiety associated with COVID-19 infection.

	**Never**	**Unlikely**	**Maybe**	**Most likely**	**Definitely**
Since last week, how often do you think about coronavirus pandemic?	27 (6%)	76 (16%)	130 (27%)	148 (31%)	100 (21%)
Since last week, how often you feel paranoid about contracting the coronavirus?	136 (28%)	71 (15%)	168 (35%)	68 (14%)	37 (8%)
Since last week, how often do you avoid partying?	47 (10%)	38 (8%)	111 (23%)	118 (25%)	165 (34%)
Since last week, how often do you avoid social contact?	52 (11%)	59 (12%)	132 (27%)	124 (26%)	114 (24%)
Since last week, how often do you avoid large meetings and gatherings?	47 (10%)	36 (8%)	97 (20%)	130 (27%)	170 (35%)
Since last week, how often have you talked to your friends about the coronavirus?	49 (10%)	66 (14%)	178 (37%)	107 (22%)	80 (17%)
Since last week, how often have you had difficulty sleeping due to worries about the coronavirus pandemic?	229 (48%)	113 (24%)	79 (16%)	24 (5%)	35 (7%)
Since last week, how often do you feel the need to buy and stock essentials at home?	233 (49%)	89 (19%)	87 (18%)	30 (6%)	32 (7%)
Since last week, how often are you afraid if anyone in your social circle reports being sick?	120 (25%)	100 (21%)	135 (29%)	59 (13%)	57 (12%)
Since last week, how often do you feel the need to use hand sanitizer, mask, or gloves?	53 (11%)	59 (13%)	91 (19%)	103 (22%)	165 (35%)
Since last week, how often do feel the need to constantly wash your hands?	36 (8%)	60 (13%)	93 (20%)	99 (21%)	183 (39%)
Since last week, how often are you worried about yourself and loved ones regarding the spread of the coronavirus?	64 (14%)	62 (13%)	122 (26%)	79 (17%)	144 (31%)
Since last week, how often does the idea of coronavirus infection freak you out leading you to make inappropriate behaviors with anyone?	192 (41%)	79 (17%)	103 (22%)	52 (11%)	45 (10%)

### Perceived Mental Healthcare Needs

**Figure 3:**
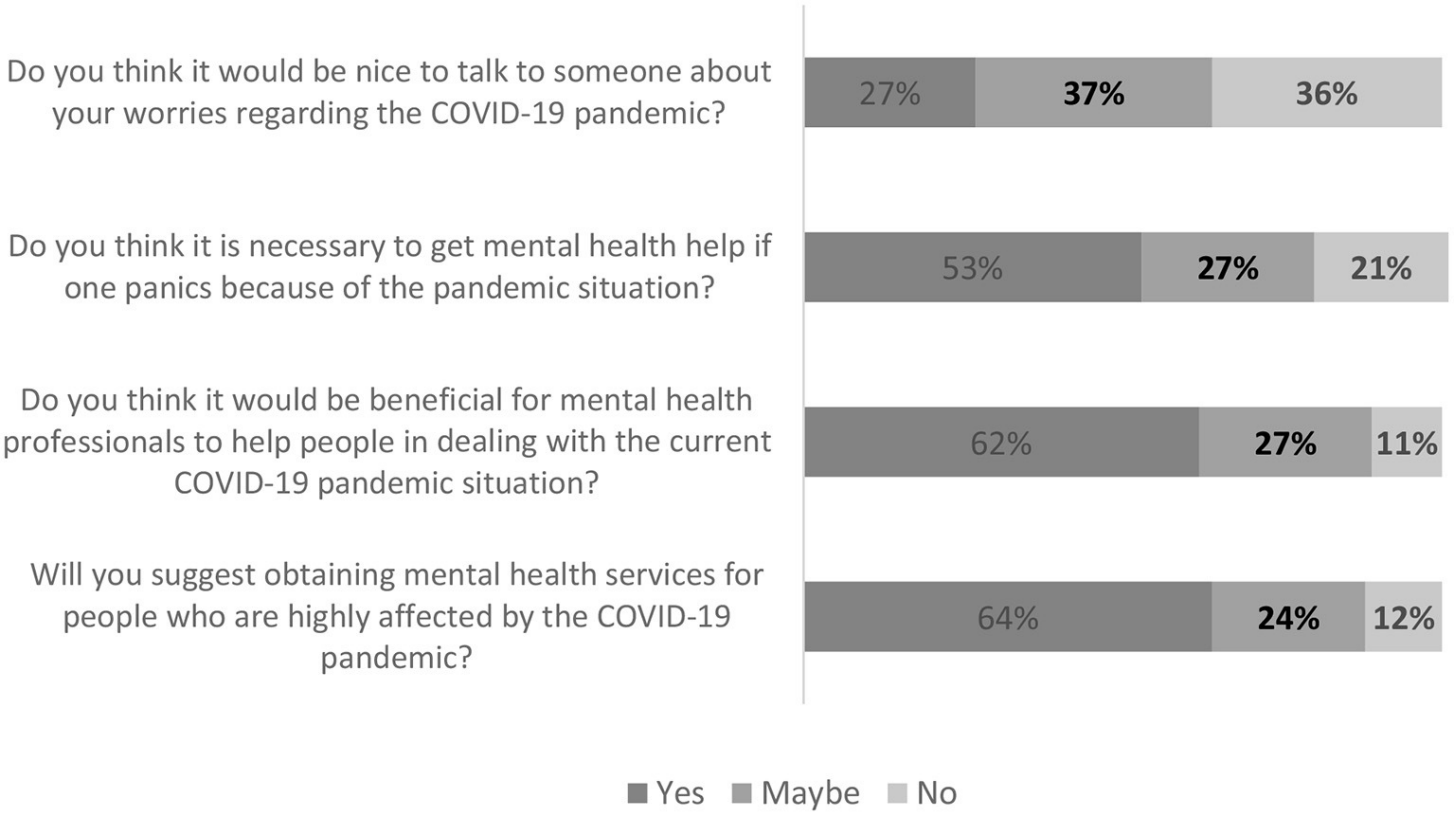
Perceived mental healthcare needs among participants.

The pandemic has been a source of psychological distress worldwide. Data presented in Table 7 and Figure 3 showed that more than 64% of participants were open to talking to a professional, family, or close friends about concerns associated with COVID-19. Most participants (80%) felt it was necessary for individuals experiencing fear and anxiety related to the pandemic to seek help from mental health professionals. Furthermore, a significant number of participants (89%) thought it would be beneficial for mental health professionals to help people deal with the current COVID-19 pandemic. Participants highly approved (88%) obtaining mental health services for people who are highly affected by the pandemic.

**Table 7:** Perceived mental healthcare needs among participants.

	**Yes**	**Maybe**	**No**
Do you think it would be nice to talk to someone about your worries regarding the COVID-19 pandemic?	129 (27%)	174 (37%)	167 (36%)
Do you think it is necessary to get mental health help if one panics because of the pandemic situation?	247 (53%)	125 (27%)	98 (21%)
Do you think it would be beneficial for mental health professionals to help people in dealing with the current COVID-19 pandemic situation?	292 (62%)	125 (27%)	53 (11%)
Will you suggest obtaining mental health services for people who are highly affected by the COVID-19 pandemic?	305 (64%)	114 (24%)	59 (12%)

## Discussion

COVID-19 continues to surge around the world, with more than 271 million confirmed cases and over 5 million deaths across nearly 200 countries (5). As the COVID-19 virus continues to evolve and develop new strains, more COVID-19 cases are expected. Scientists are working tirelessly to learn more about emerging variants, their spread, and whether vaccines will be protective against them. The pandemic is ongoing, therefore, the public must be informed and updated with any new information and developments related to the virus and its variants. As was seen in previous epidemics and pandemics, the populace faces many challenges and hardships. Lack of awareness and accurate information can lead to attitudes and behaviors which work against the goal of slowing the spread of the virus and ending the pandemic. Therefore, this study sought to examine individuals' knowledge and attitudes toward COVID-19. In addition, the study measured levels of anxiety caused be COVID-19 and examined the need for mental health services in Saudi Arabia.

The level of knowledge seemed to be moderately high among study participants, which is similar to other studies conducted among educated people in India (16), Ethiopia (16, 17) and other countries (18–20). More specifically, the knowledge level found in this study is congruent with other studies conducted in the Saudi population (21–23). The study provides additional evidence to earlier findings. It seems that government media coverage in Saudi Arabia kept the public highly informed about the pandemic. It is worth mentioning that the data collection was not during the early stages of the pandemic, and hence higher level of awareness is expected. Furthermore, non-government outlets and social media platforms increased their coverage as the number of COVID-19 cases increased. The media, in general, updated and informed the public about COVID-19 with a focus on the publications that adversely affect people's life and health. When the media reporting negative stories constantly, the audience most likely to carry negative attitude toward the COVID-19 pandemic (24). It is known that attitude is associated with a person's response to the surrounding stimuli. The study participants showed a significant increase in following protective measures such as wearing face coverings, washing hands, social distancing, and self-quarantining if they felt ill (25). These behaviors indicate high concern of contracting COVID-19. Thus, a negative attitude is expected as media coverage overwhelmingly showed the negative side of the pandemic on individuals and public health. It could play protective roles in pandemic situations.

As COVID-19 affects a large proportion of society, a variety of preventive measures were implemented at the personal and societal level, such as limiting daily activities and social interaction. Removing people from their work, education, and social activities, though beneficial to slowing virus transmission, took a toll on mental health, causing individuals to experience panic, stress, confusion, and sleep problems. Additionally, people may become worried about unexpected health problems, causing them additional stress, and misinformation about the pandemic can lead to fear and panic. Approximately half of study participants often feel worried about their own risk of becoming infected by the virus, as well as family and friends. The results of this study suggested that COVID-19 increased anxiety and stress levels among participants. These findings are consistent with those found in the literature in the last few months (26–31). COVID-19 can be the cause of high anxiety, especially among the elderly or those with chronic health problems (32). Hence, it is likely that the COVID-19 pandemic has contributed to poor mental health outcomes. For example, the U.S. Census Bureau Household Pulse Survey reported a significant increase of anxiety and depression symptoms in May 2020 (33) compared to the first 3 months of 2019. The world has passed through an historic pandemic and the evidence has shown detrimental effects on mental health (34, 35). COVID-19 is unprecedented in terms of economic lockdown and social isolation, as is its significant impact on mental health in all age groups (36).

In this study, over half of the participants thought it would be nice to talk to someone about their worries regarding the COVID-19 epidemic. Most study participants approved of providing mental health services to those affected by the pandemic. However, access to mental health services was very limited in Saudi Arabia—due to stigma about mental illness—prior to the pandemic and could be inaccessible during and after the pandemic. Therefore, mental health problems could be expected to continue and worsen with time if no psychological intervention were provided. Thus, it is recommended that individuals be encouraged to obtain mental health services, and that these services be made accessible for anyone who requires them. Online mental health consultations are another option, which might reduce any stigma resulting from visiting mental health clinics.

Undoubtedly, there is a need to focus more on strategies to help and support those at risk of developing mental health issues. Health education is critical at this point to reach out to community members and provide mental health guidelines. Enhancing access to psychological interventions is important at this time with new approaches such as web-based intervention and telepsychiatry. This cannot be done without collaboration and support from Saudi health authorities. In addition, health authorities must regularly provide updates regarding the pandemic, including effective prevention measures, vaccines, and new emerging threats, and fight misinformation and rumors, especially about the safety and efficacy of the COVID-19 vaccines. Accurate health information could counter negative thinking and buffer anxiety, stress, and depression.

The study limitation includes a sampling bias, as the study targeted only individuals who had access to the Internet, used What's App, and were linked directly or indirectly to the study investigators. This was evidenced by the fact that the median age is 23 years. Given that the sample size is also small, it is imperative that the findings cannot be generalized to the whole of Saudi society. In addition, causal inferences cannot be made with a cross-sectional study design.

## Conclusion

The findings of this study reveal that most people have developed a satisfactory level of knowledge about the coronavirus and possible preventive measures to limit its transmission. However, respondents experienced worries and fears about the virus, particularly about being infected. The perceived threat of infection could lead adverse psychological outcomes such as health anxiety, stress, and depression. Therefore, there is an urgent need for more health education and awareness programs to make individuals cautious not to panic about COVID-19 and its variants. Ongoing educational programs may alleviate negative attitudes and encourage constructive preventive and restorative practices.

## Data Availability Statement

The raw data supporting the conclusions of this article will be made available by the authors, without undue reservation.

## Ethics Statement

Ethical review and approval was not required for the study on human participants in accordance with the local legislation and institutional requirements. The patients/participants provided their written informed consent to participate in this study.

## Author Contributions

All authors contributed equally to the conception and design, acquisition of data, or analysis and interpretation of data, took part in drafting the article or revising it critically for important intellectual content, gave final approval of the version to be published, and agreed to be accountable for all aspects of the work.

## Conflict of Interest

The authors declare that the research was conducted in the absence of any commercial or financial relationships that could be construed as a potential conflict of interest.

## Publisher's Note

All claims expressed in this article are solely those of the authors and do not necessarily represent those of their affiliated organizations, or those of the publisher, the editors and the reviewers. Any product that may be evaluated in this article, or claim that may be made by its manufacturer, is not guaranteed or endorsed by the publisher.
